# Systematic analysis of randomised controlled trials of Chinese herb medicine for non-alcoholic steatohepatitis (NASH): implications for future drug development and trial design

**DOI:** 10.1186/s13020-023-00761-5

**Published:** 2023-05-19

**Authors:** Xianwen Chen, Junnan Shi, Yunfeng Lai, Yan Xue, Carolina Oi Lam Ung, Hao Hu

**Affiliations:** 1 State Key Laboratory of Quality Research in Chinese Medicine, Institute of Chinese Medical Sciences, University of Macau, Taipa, Macao China; 2 School of Public Health and Management, Guangzhou University of Chinese Medicine, Guangzhou, China; 3 Department of Public Health and Medicinal Administration, Faculty of Health Sciences, University of Macau, Taipa, Macao China

**Keywords:** Non-alcoholic steatohepatitis, Traditional Chinese medicine, Randomised controlled trial, Clinical trial design

## Abstract

**Background:**

Non-alcoholic steatohepatitis (NASH) is a liver disease currently lacking an approved therapy, resulting in significant clinical demand. Traditional Chinese medicines (TCMs) have been commonly used to manage NASH. This study aimed to systematically analyse the randomised controlled trials (RCTs) using TCMs for NASH management.

**Methods:**

A systematic literature review was performed by following PRISMA guidelines 2020 in six electronic databases: PubMed, Web of Science, Scopus, Embase, the Cochrane Library, and China National Knowledge Infrastructure, from inception until August 2022. RCTs using TCMs for NASH were included in the analysis, irrespective of language or blinding.

**Results:**

112 RCTs were included in this review, with 10,573 NASH participants. 108 RCTs were conducted in China, and 4 RCTs were in other countries. Herbal medicine decoction was the major dosage form used for treating NASH (82/112). 11 TCMs products have been approved for NASH treatment (8 in China, 2 in Iran, and 1 in Japan). Classic prescriptions, such as “Huang Lian Jie Du decoction”, “Yin Chen Hao decoction”, and “Yi Guan Jian” were used in some studies. The TCMs treatment of NASH involved the use of 199 different plants, with the top 5 herbs being Salviae Miltiorrhizae Radix Et Rhizoma, Alismatis Rhizoma, Bupleuri Radix, Poria, and Curcumae Radix. “Salviae Miltiorrhizae Radix Et Rhizoma + Bupleuri Radix/Alismatis Rhizoma” were the mostly common drug-pair in the herbs network analysis. Nowadays, “Bupleuri Radix/Alismatis Rhizoma + Atractylodis Macrocephalae Rhizoma” are increasingly applied in herbal formulas for NASH. Based on the PICOS principles, the included studies varied in terms of the population, intervention, comparator, outcomes, and study design. However, some studies reported unstandardised results and failed to report diagnostic standards, inclusion or exclusion criteria, or sufficient patient information.

**Conclusion:**

Adopting Chinese classic prescriptions or drug-pair may provide a basis for developing new drugs of NASH management. Further research is needed to refine the clinical trial design and obtain more convincing evidence for using TCMs to treat NASH.

**Supplementary Information:**

The online version contains supplementary material available at 10.1186/s13020-023-00761-5.

## Background

Non-alcoholic steatohepatitis (NASH), as an inflammatory subtype of non-alcoholic fatty liver disease, is associated with the progression of the severe liver illness, such as liver fibrosis, hepatocellular carcinoma, the occurrence of cirrhosis, liver transplant, and death [1–3]. Between 2016 and 2030, the prevalence of NASH is projected to increase by up to 56% in China, France, Germany, Italy, Japan, Spain, the United Kingdom, and the United States [4]. The corresponding prevalence of NASH-related comorbidities was: 82% with obesity; 82% with hyperlipidemia; 76% with metabolic syndrome; 70% with hypertension; 48% with type 2 diabetes mellitus[5]. Additionally, NASH raises the risk of cardiovascular disease, cancer, and chronic renal disease [6, 7]. Therefore, NASH’s high prevalence and severe complications pose a significant societal burden. According to an assessment of the financial impact of NASH in five European nations and the United States, the average direct medical, direct non-medical, and indirect expenses per patient per year were €2763, €4917, and €5509, respectively [8]. In Hong Kong, the average yearly cost of NASH per person was $257 [9].

There is a significant clinical need for a medicine to treat NASH, yet none has been licensed [10]. The two primary therapies for NASH are pharmacological treatments and weight loss or weight control techniques. Drugs that regulate glucolipid metabolism, prevent oxidative stress or inflammatory reactions, have anti-fibrotic properties, and regulate gut flora are also used to manage NASH [11, 12]. These approaches aim to address the underlying pathophysiological mechanisms that contribute to the development and progression of NASH. Despite some promising results in clinical trials, further research is still needed to identify safe and effective treatments for this condition.

Traditional Chinese medicines (TCMs) is increasingly employed in managing or treating liver problems and is based on tailored treatment procedures [13]. The use of TCMs for treating NASH has been shown to be effective in leading to a variety of pathological changes, including improved hepatic lipid metabolism, decreased liver inflammation, reduced fibrosis, and ameliorated intestinal flora. The potential of TCMs for NASH has been validated by current pharmacological methods in some classic traditional Chinese prescriptions [12]. A growing number of clinical trials or research studies using TCMs to treat NASH have been conducted in recent years. However, the TCMs used in various studies were unique. Notably, a systematic analysis of traditional Chinese medicine prescriptions is lacking. Furthermore, there are still flaws in the clinical trial design, even though evaluating the quality of randomised controlled trials (RCTs) of TCMs generally found a minimal risk of bias [12, 14].

Therefore, this study aimed to systematically analyse the RCTs using TCMs for NASH management. These findings will aid in advancing TCMs medication research for NASH and generating suggestions for future TCMs RCTs for NASH.

## Methods

### Literature search and screen

This systematic analysis was performed following PRISMA guidelines 2020 [15]. The literature research was conducted in six electronic databases: PubMed, Web of Science, Scopus, Embase, the Cochrane Library, and China National Knowledge Infrastructure, from inception up to August 2022.

The related Mesh terms or synonyms were also searched (as shown in Table 1). Two researchers separately finished the screening. We conducted two rounds of literature screening to identify RCT using TCMs to treat NASH patients. In the first round, according to study titles and abstracts, we initially excluded the following studies: (1) the language is neither English nor Chinese; (2) Animal studies, quasi-randomised control trials, non-randomised trial, pharmacodynamics investigations, and in vitro studies; (3) Reviews, commentaries, letters, conference abstracts, notes, editorial material, guidelines. The second round involved a full text review in screening for any RCTs using TCMs on NASH. References of included publications were also checked.

**Table 1:** Search term identifiers

Category	Entry term in English	Entry term in Chinese
Non-alcoholic steatohepatitis	NASH	非酒精性脂肪肝炎
	Non-alcoholic steatohepatitis	非酒精性脂肪性肝炎
	Nonalcoholic steatohepatitides	
	Steatohepatitides, nonalcoholic	
	Steatohepatitis, non-alcoholic	
TCMs	Phytotherapy	中醫
	Herbal medicine^a^	中藥
	Plant preparation^a^	草藥
	Chinese medicine^a^	
	Complementary medicine^a^	
	“Drugs, Chinese herbal” Mesh)	
	“Medicine, Chinese traditional” (Mesh)	
	“Medicine, traditional” (Mesh)	
	“Plant preparations” (Mesh)	
	Medicinal plant^a^	
	Plant medicinal product^a^	
	Herb^a^	
RCT	Clinical	隨機 AND 對照 AND 臨床試驗
	Trial^a^	隨機 AND 對照 AND 臨床研究
		隨機 AND 對照 AND 臨床觀察

AND retrieves results that include all the search terms

^a^Including but not limited to

### Data collection and management

The TCMs medication and clinical research design were included in a preset data extract form (Table 2). About the medication, different drug types and specific materials in each formula maybe collected. Chinese herbal formulas in this research represented using different herbals for treating NASH, which included various dosage forms, such as decoction, granules, capsules, tablets and pills. And as for the clinical research design, the data extract content was developed in accordance with PICOS principles [16].

**Table 2:** Data collection

Data category	Content
Medication	Medication type
	Herbal formula, dosage form
	Single herb extract, dosage form
	Herbs
	The specific materials in each formula
Clinical research design	Patient enrollment
	Source: single hospital, multiple hospital
	Characteristics: country, age, history of NASH, comorbidity, visit type
	Sample size
	Diagnostic criteria
	Interventions
	Other intervention besides medication
	Time period: trial duration and follow-up period
	Comparator
	Other intervention besides medication
	Time period: trial duration and follow-up period
	Outcome measures
	Primary outcome: overall clinical efficacy rate, hepatic function outcomes, blood lipids profiles, Radiological response improvement
	Secondary outcome: TCMs syndrome scores, blood sugar, liver fat, indexes of inflammation and tumor necrosis factor, other indicators
	Efficacy evaluation criteria and results
	Adverse effects
	Clinical research design
	Single-arm/two-arms/three-arms

The TCMs syndrome score is a rating system used to assess patient symptoms such dry mouth, bitter eyes, dry eyes, bleeding gums, sleeplessness and nightmares, abdominal distension, loss of appetite, weariness, lack of appetite, hypochondriac pain, waist and knee pain, urine and stool, etc. A score of 0 points, 1 point, 2 points, or 3 points would indicate that the symptoms were "no," "mild," "moderate," or "severe," respectively [17]. Therapeutic effect index = (pre-treatment TCMs symptom score − post-treatment score)/pre-treatment TCMs symptom score × 100% [17].

### Data analysis

Data extraction based on the requirements of Table 1 was carried out independently by two researchers. Any disagreements between the two researchers were resolved through discussion and with the help of a third researcher. Descriptions of data were completed by Microsoft^®^ Excel^®^ 2019MSO. The Gephi (https://gephi.org/) software was used to visualise the network layout of different herbs in different formulas [1]. In the network diagram, each node represents a herb, and each edge represents the relationship between two herbs. This approach allowed for a visual representation of the relationships between different herbs and their usage in various formulas, which can provide insights into potential synergistic effects and aid in developing new herbal formulations.

## Results

### Search result

**Fig. 1:**
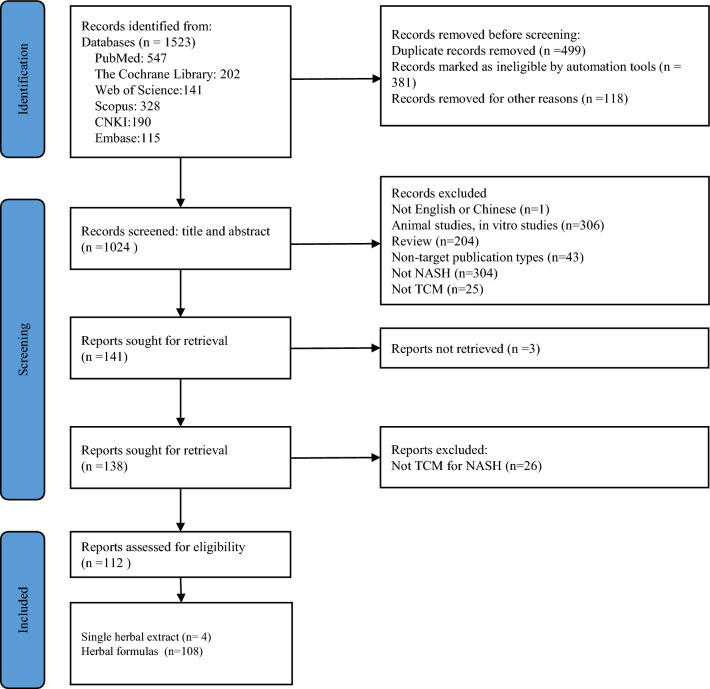
PRISMA flow-chart of study selection

Of the 1523 references identified through the search strategy, 499 duplicate articles were excluded. After two rounds of screening, 112 studies were finally included in this review (Fig. 1). 106 of the 112 publications covered in this review were published in Chinese, while 6 were published in English.

**Fig. 2:**
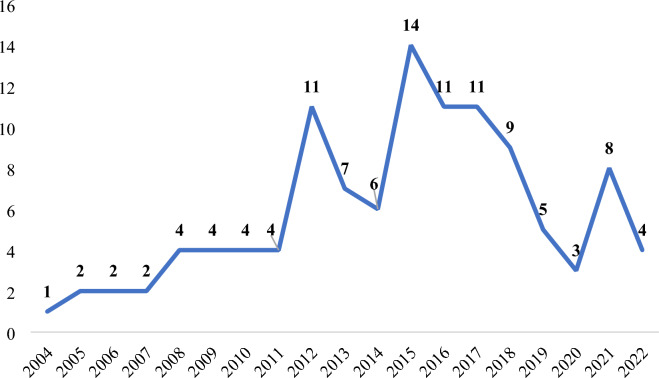
The publication trend of TCM for NASH

All the studies were published between 2004 and 2022. The overall trend of article publication is on the rise and was more concentrated between 2011 and 2017 (Fig. 2).

### Medication of TCMs for NASH

#### Medication types

According to the unique composition, there are four RCTs on single herb extract [19–22] and 108 RCTs on Chinese medicine formulas have been included [23–130] (as shown in Table 3). Various TCMs dosage forms were used in the management of NASH across the included RCTs. Among the 82 RCTs, herbal medicine decoctions were applied. Out of these, 31 RCTs used herbal medicine decoction in combination with western medicine, while 50 RCTs used only herbal medicine decoction in the test group. One study used herbal medicine decoction combined with Chinese patent medicine [83]. None of the herbal medicine decoction used in the studies has been approved.

**Table 3:** Medication of included trials for treating NASH

Medication	Trials number
Herbal formulas	108
Herbal medicine decoctions (HMD)	82
Only HMD in test group	50
HMD combined with western medicine (WM) in test group	31
HMD combined with Chinese patent medicine	1
Herbal medicine granules (HMG)	13
Only HMG in test group	2
HMG combined with WM in test group	11
Herbal medicine capsules (HMC)	9
Only HMC in test group	5
HMC combined with WM in test group	4
Herbal medicine tablets (HMT)	2
Only HMT in test group	1
HMT combined with WM in test group	1
Only herbal medicine medicine mixture in test group	1
Only herbal medicine medicine pill in test group	1
Single herb extract	4
Only herb extract tablets in test group	3
Only herb extract Capsules in test group	1

Thirteen RCTs used herbal medicine granules, out of which 11 trials used herbal medicine granules in combination with western medicine [25, 30, 43, 47, 48, 53, 71, 78, 107, 111, 114] and 2 trials used only herbal medicine granules in test group [44, 59]. It worth noting that Shen Ge granules [43] have been approved as a TCMs hospital prescription in Shanghai, and the approved number is Z20220016000.

Nine RCTs used herbal medicine capsules for NASH in test group, out of which four trials used herbal medicine capsules combined with western medicines [33, 40, 89, 121]. All of these capsules have been approved in China, including Jiang Zhi Tong Mai capsules (National license number: Z20026429) [33], Shen Ze Shu Gan capsules (National license number: Z20130022) [40], Fu Zheng Hua Yu capsules (National license number: Z20020074) [89], and Zhi Bi Tai capsules (National license number: Z51022196) [121]. 5 trials used only TCMs capsules in test group [55, 87, 88, 108, 125], among which Da Huang Li Dan capsules (National license number: Z20025609) [55], Zhi Gan capsules (Henan license number: Z05010566) [108] have been approved in China.

Two RCTs used herbal medicine tablets in the test group, one of which was TCMs combined with western medicine [52]. The other trial used only TCM tablet [126], which have been approved in Japan and have a history of more than 28 years (trade name: kyotsujigyo, https://kyotsujigyo.net/). Additionally, one trial used herbal medicine mixture in test group [98], and one trial used herbal medicine pills (listed in Beijing as a TCMs prescription in medical institution with approval number Z20190022000) [112].

Noteworthy, there were 3 RCTs that used the hospital preparations of TCMs, which included Shen Ge granules [43], Zhi Gan capsules [108], and Jian Pi Shu Gan pill [112]. Hospital preparations, also known as medical institution preparations, refer to preparations that medical institutions make according to the clinical needs of the unit. They are approved by the provincial, autonomous regions, or municipalities directly under the Central Drug Supervision and Administration Department for hospital consultation and fixed prescription preparations for patients. These preparations belong to the hospital's own pharmaceuticals.

From the results of drug generic name statistics, the medication of control group of included RCTs for NASH is dominated by chemical drugs (as shown in Table 4), and the drugs most often used as controls are Polyene Phosphatidylcholine Capsules, Silybin Meglumine Tablets or granules, and Tiopronin Tablets. Secondly, biological drugs and proprietary Chinese medicines are also used as control drugs.

**Table 4:** Control group medication of included trials for treating NASH

Control group medication	Trials number
Only chemical medicine	89
Polyene phosphatidylcholine capsules	41
Silybin meglumine tablets/granules	14
Tiopronin tablets	6
Ursodeoxycholic acid tablets	4
Vitamin E capsule	3
Diammonium glycyrrhizinate enteric-coated capsules	2
Reduced glutathione tablets/injection	3
Others	4
Multi-chemical medicine combination	12
Chinese patent medicine	6
Danning tablets	2
Hugan tablets	3
Yiganning tablets	1
Biological products	3
Bifidobacterium triptans capsules	1
Bacillus licheniformis capsules	1
Bacillus subtilis duplex enteric capsules	1
Chinese patent medicine combined with chemical medicine	2
Dangfei yiganning capsules + ursodeoxycholic acid tablets	1
Silibinin tablets + polyene phosphatidylcholine capsules	1
Liver protection or enzyme reduction	1
Placebo	7
Non-pharmacological therapy (diet/exercise)	3
Self-comparison in before and after	1

### Herbal formulas

#### Herbal formulas information

To determine the composition of herbs in prescriptions, we analysed the prescription information from the 108 included RCTs that used herbal formulas for NASH. Of these, 69 trials utilised herbal formulas alone, while 39 trials chose herbal formulas in conjunction with other medications. Additionally, one study found that the herbal formulas it looked at were not superior to western medicines [71], while another study discovered that the effects of the herbal formulas it looked at were similar to those of western medicines [98].

There were 21 RCTs based on the Chinese traditional prescriptions to treat NASH. The information about composition, functions and resources of Chinese traditional prescriptions that are applied in the included RCTs is shown in Table 4. The remaining research used various prescriptions by adding other herbs based on traditional Chinese medicine fomulas. Based on the "Catalogue of Traditional Chinese Medicine Classical Prescription (First Batch)"of National Administration of Traditional Chinese Medicine in China, we analysed all prescription compositions. Two RCTs included the composition of "Ling Gui Zhu Gan Decoction" [51], and one study also included the composition of "Yi Guan Decoction" [74].

The formulas of some RCTs did not mention the referenced prescriptions, but their herbal composition reflected the composition of some traditional Chinese medicine classical prescriptions. For instance, 21 trials contained Alismatis Rhizoma and Atractylodis Macrocephalae Rhizoma, which are the composition of the classical prescriptions “Ze Xie Decoction”. Additionally, 5 trials contained Astragali Radix and Angelicae Sinensis Radix Angelicae, which are the composition of the classical prescriptions “Dang Gui Bu Xue Decoction”.

#### Herbs analysis

**Fig. 3:**
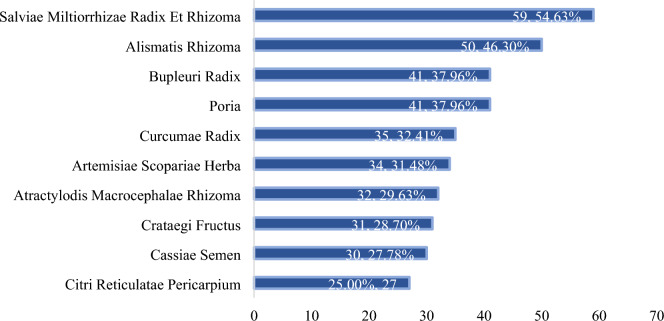
Top 10 Chinese herbs in 107 trials using Chinese medicine formula

Totally 199 Chinese herbs were employed in 108 RCTs, with Salviae Miltiorrhizae Radix Et Rhizoma being the most frequently used herb, followed by Alismatis Rhizoma, Bupleuri Radix, Poria, Curcumae Radix, Artemisiae Scopariae Herba, Atractylodis Macrocephalae Rhizoma, Crataegi Fructus, Cassiae Semen, and Citri Reticulatae Pericarpium, in descending order (n% = n/108 × 100%) (Fig. 3).

#### Herbs network analysis

**Fig. 4:**
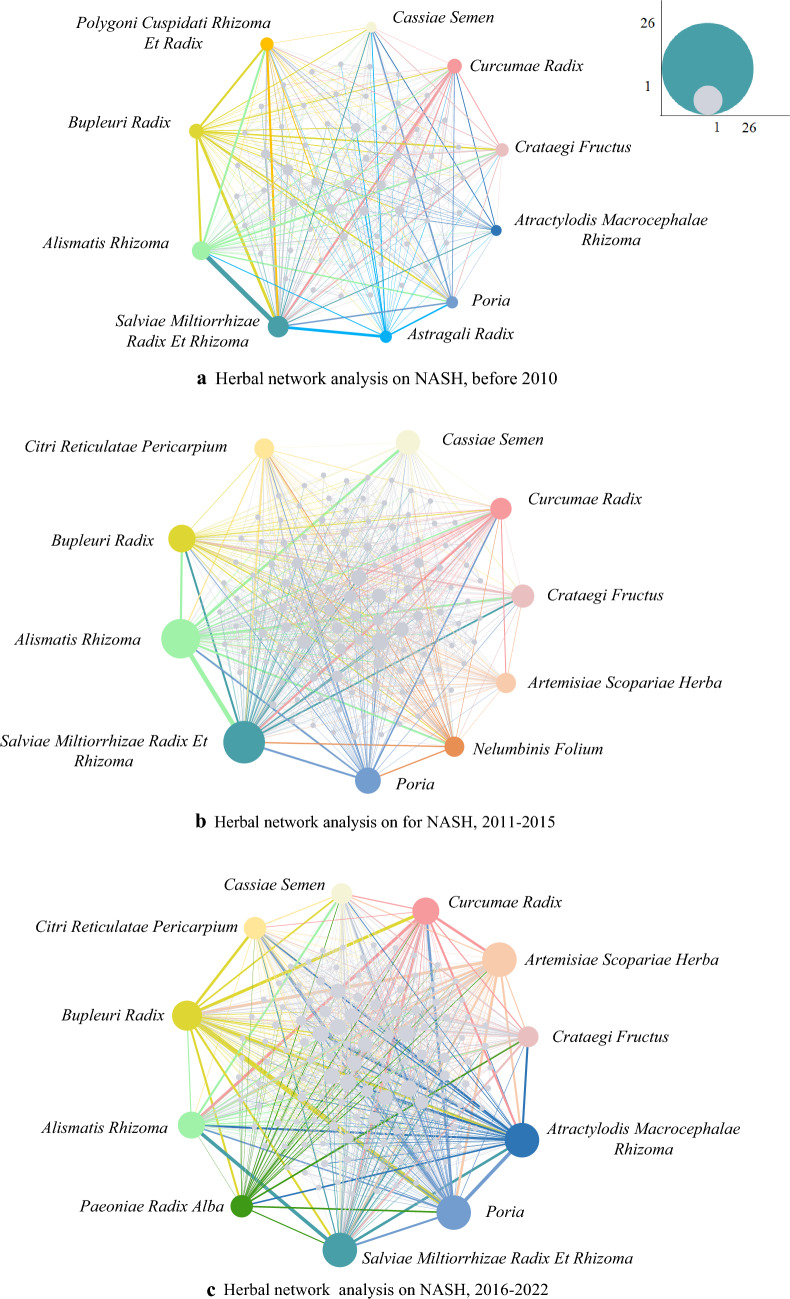
The dynamic network of Herbs, **a** before 2010, **b** from 2011 to 2015, **c** after 2015. The network diagram in this study represents the presence of herb pairs in prescriptions for NASH. Node size is scaled to the frequency of Chinese herbs of each TCMs prescriptions. The thickness of edges represents the frequency of using there two herbs on one prescription, which is counted by the number of the appearance frequency of each herb between two prescriptions. Top 10 materials were labeled in different color, others were in gray color. Nodes in  denote Salviae Miltiorrhizae Radix Et Rhizoma. Nodes in  denote Alismatis Rhizoma. Nodes in  denote Bupleuri Radix. Nodes in  denote Poria. Nodes in  denote Curcumae Radix. Nodes in  denote Artemisiae Scopariae Herba. Nodes in  denote Atractylodis Macrocephalae Rhizoma. Nodes in  denote Cassiae Semen. Nodes in  denote Astragali Radix. Nodes in  denote Citri Reticulatae Pericarpium. Nodes in  denote Polygoni Cuspidati Rhizoma et Radix. Nodes in  denote Paeoniae Radix Alba. Nodes in  denote Nelumbinis Folium. Nodes in  denote Crataegi Fructus

Network analysis was applied to explore the relationship between Chinese herbs used in treating NASH in different years. In Fig. 4, Salviae Miltiorrhizae Radix Et Rhizoma (SMRER), Alismatis Rhizoma (AR), Bupleuri Radix (BR), Curcumae Radix (CR), Crataegi Fructus (CF), and Poria remained in the top 10, with a gradual increase in the use of Atractylodis Macrocephalae Rhizoma (AMR) and Artemisiae Scopariae Herba (ASH). The Chinese herbs used to treat NASH have changed over different periods, with an increase in the number of herbs used and the complexity of their relationships. However, "SMRER and AR" and "SMRER and BR" remained closely interconnected throughout the different time periods.

The network diagram in this study represents the presence of pairs of herbs in prescriptions for the treatment of NASH, with nodes representing individual Chinese medicinal materials and edges indicating co-occurrence in the same prescription. The size of the nodes indicates the frequency of the Chinese herbs, while the width of the edges represents the frequency of co-occurrence.

Figure 4a displayed 67 nodes representing 67 Chinese herbs mentioned in prescriptions for NASH, with a total of 604 edges indicating 763 pairs of herbs appearing together. Before 2010, SMRER appeared 13 times (7.78%) and was closely related to AR, PCRER, and BR.

Figure 4b showed 128 nodes and 1382 edges, indicating 128 herbs appearing in pairs 1382 times. Between 2011 and 2015, SMRER appeared 26 times (6.65%), while PCRER and BR did not appear in the top 10. The most commonly used pairs during this period were "SMRER and BR" (n = 7), "SMRER and CF" (n = 7), and "SMRER and AR" (n = 6).

Figure 4c contained 138 nodes and 1577 edges, indicating 1577 pairs of Chinese herbs. After 2015, AMR, SMRER, and ASH all appeared 20 times (4.41%), and PRA reached the top 10 for the first time with a frequency of 12 (2.64%). The high-frequency pairs during this period were "PRA and SMRER" (n = 4), "SMRER and Poria" (n = 5), "AMR and CR", and "AMR and BR". These pairs differed from those in previous periods.

#### Single herbal extract

Four RCTs investigated the effects of herbal extracts on NASH. The herbal extract studied were: Cumin [19], Phyllanthus urinaria [20], Cynara scolymus [21], and Silymarin [22]. Cumin was in capsule form and listed in Iran at https://en.goldaruco.com/product/livergol-tablet/. Phyllanthus urinaria, Cynara scolymus, and Silymarin (listed in Iran, https://barijessence.com/en/) were all in tablets form. The trial of Phyllanthus urinaria was conducted in Hong Kong, and the other three were conducted in Iran.

Two studies found that a single herbal extract was not significantly more beneficial than a placebo [19, 20]. Two trails reported that single herb extract improved the symptom of NASH [21, 22], while two trails reported no significant differences between herbal extract group and control group [19, 20].

### Clinical research design

#### Overview of the clinical research design

As shown in Table 5, 10,573 participants were included in this study. Among them, 10,341 participants in 108 RCTs were enrolled in China, 224 participants in three RCTs were enrolled in Iran [19–22], and eight participants in one RCT were enrolled in Japan [126]. The age range of participants was 16–76 years.

**Table 5:** Traditional Chinese formulas information of included RCTs

No	Formula name	Composition (Latin name)	Action	Formula resources	References
1	Er Chen decoction	Pinelliae Rhizoma, Poria, Citri Exocarpium Rubrum, Glycyrrhizae Radix Et Rhizoma	Removing dampness, resolving phlegm and regulating the spleen and stomach	*“Prescriptions of the Bureau of Taiping People’s Welfare Pharmacy”*(太平惠民和劑局方)	Wang et al.* [124]
	(二陳湯)		燥濕化痰, 理氣和中		Chen et al.* [74]
2	Bu Bi Wei Xie Yin Huo Sheng Yang Decoction*	Bupleuri Radix, Glycyrrhizae Radix Et Rhizoma Praeparata Cum Melle, Astragali Radix, Atractylodis Rhizoma, Notopterygii Rhizoma Et Radix, Cimicifugae Rhizoma, Ginseng Radix Et Rhizoma, Coptidis Rhizoma, Gypsum Fibrosum	Regulating the spleen and stomach, purging the pathogenic fire	*“Treatise on the Spleen and Stomach”(脾胃論)*	Wang et al.* [118]
	(補脾胃瀉陰火升陽湯)		補脾升陽瀉火		
3	Yin Chen Hao Decoction	Artemisiae Scopariae Herba, Gardeniae Fructus, Rhei Radix Et	Clearing heat, promoting dampness, and removing jaundice	*“Treatise on Exogenous Febrile Disease”(傷寒論)*	Chen et al.* [73]
	(茵陳蒿湯)	Rhizoma	清熱, 利濕, 退黃		Tang et al.* [65]
					Liu et al. [56]
					Lin et al.* [47]
					Lan et al.* [38]
4	Huang Lian Jie Du decoction	Coptidis Rhizoma, Scutellariae Radix, Phellodendri Chinensis	Clearing away heat and reducing fire	*“Prescriptions for Emerent Reference”(肘後備急方)*	Liu et al. [49]
	(黃連解毒湯)	Cortex, Gardeniae Fructus	清熱瀉火		Wang et al. [26]
5	Sheng Jiang decoction	Codonopsis Radix, Astragali Radix, Atractylodis	Regulates the liver and spleen and promotes digestion	*“Yi Xue Zhong Zhong Can Xi Lu”*	Feng et al. [77]
	(升降湯)	Macrocephalae Rhizoma, Citri Reticulatae Pericarpium, Magnoliae Officinalis Cortex, Galli Gigerii Endothelium Coreneum, Anemarrhenae Rhizoma, Paeoniae Radix Alba, Cinnamomi Ramulus, Chuanxiong Rhizoma, Zingiberis Rhizoma Recens	調節肝脾, 促進消化	*(醫學衷中參西錄)*	Zou et al. [42]
6	Four Gentlemen Decoction	Ginseng Radix Et Rhizoma, Atractylodis Macrocephalae Rhizoma, Poria, Glycyrrhizae Radix Et Rhizoma	Relieving fatigue and strengthening the spleen	*“Prescriptions of the Bureau of Taiping People’s Welfare Pharmacy”(太平惠民和劑局方)*	Chen et al. [74]
	(四君子湯)		益氣健脾		
7	Yi Guan Decoction	Glehniae Radix, Ophiopogonis Radix, Angelicae Sinensis Radix, Rehmanniae Radix, Lycii Fructus, Toosendan Fructus	Regulate liver function	*“Xu Mingyi Lei ‘an”(續名醫類案)*	Chen et al. [74]
	(一貫煎)		滋陰疏肝		
8	Xiao Jian Zhong Decoction	Cinnamomi Ramulus, Glycyrrhizae Radix Et Rhizoma Praeparata Cum Melle, Jujubae Fructus, Paeoniae Radix Alba, Zingiberis Rhizoma Recens, Maltose	Warming the middle and tonifying the deficient, harmonising the inner	*“Treatise on Exogenous Febrile Disease”(傷寒論)*	Chen et al. [74]
	(小建中湯)		溫中補虛, 和裡緩急		
9	Ge Xia Zhu Yu Decoction	Angelicae Sinensis Radix, Persicae Semen, Glycyrrhizae Radix Et Rhizoma, Carthami Flos, Wu Ling Zhi, Chuanxiong Rhizoma, Moutan Cortex, Paeoniae Radix Rubra, Linderae Radix, Corydalis Rhizoma, Cyperi Rhizoma, Aurantii Fructus	Promoting blood circulation and eliminating blood stasis, breaking down symptoms and eliminating nodules	*“Corrections on the Errors of Medical Works”*	Chen et al. [74]
	(膈下逐瘀湯)		活血逐瘀, 破症消結	*(醫林改錯)卷上*	
10	Wu Mei Pill	Mume Fructus, Asari Radix Et Rhizoma, Zingiberis Rhizoma, Coptidis Rhizoma, Aconiti Lateralis Radix Praeparata, Angelicae Sinensis Radix, Zanthoxyli Pericarpium, Cinnamomi Ramulus, Phellodendri Chinensis Cortex, Ginseng Radix Et Rhizoma	Promoting vitality, promoting correctness, eliminating fat and removing phlegm	*“Treatise on Exogenous Febrile Disease”(傷寒論)*	Zhang et al.* [72]
	(烏梅丸)		益氣扶正, 消脂去痰		
11	Jia Wei Bao He Pill	Atractylodis Macrocephalae Rhizoma, Poria, Citri Reticulatae Pericarpium, Magnoliae Officinalis Cortex, Aurantii Fructus Immaturus, Aurantii Fructus, Cyperi Rhizoma, Massa Medicata Fermentata, Hordei Fructus Germinatus, Pinelliae Rhizoma Praeparatum	Promoting blood circulation and eliminating blood stasis, breaking down symptoms and eliminating nodules	*“Shoushibao Yuan”*	Tian et al.* [66]
	(加味保和丸)		活血逐瘀, 破症消結	*(壽世保元)*	
12	Shen Ling Bai Zhu Powder	Nelumbinis Semen, Coicis Semen, Amomi Fructus, Platycodonis Radix, Lablab Semen Album, Poria, Ginseng Radix Et Rhizoma, Glycyrrhizae Radix Et Rhizoma Praeparata Cum Melle, Atractylodis Macrocephalae Rhizoma, Dioscoreae Rhizoma	Benefiting vital energy, strengthening the spleen, penetrating dampness and stopping diarrhea	*“Prescriptions of the Bureau of Taiping People’s Welfare Pharmacy”(太平惠民和劑局方)*	Dai et al. [52]
	(參苓白術散)		益氣健脾, 滲濕止瀉		
13	Bao He Pill	Crataegi Fructus, Massa Medicata Fermentata, Pinelliae Rhizoma, Poria, Citri Reticulatae Pericarpium, Raphani Semen, Forsythiae Fructus	Promotes digestion, dissolves phlegm, dispels fatigue and breaks stagnation	*“Danxi’s Mastery of Medicine”(丹溪心法)*	Zhan et al.* [97]
	(保和丸)		消食和胃, 化痰祛疲破滯		
14	Chai Shao Liu Jun Zi Decoction	Codonopsis Radix, Atractylodis Macrocephalae Rhizoma, Poria, Glycyrrhizae Radix Et Rhizoma, Paeoniae Radix Alba, Bupleuri Radix, Ramulus Uncariae Sinensis Cum, Pinelliae Rhizoma, Citri Reticulatae Pericarpium	Strengthening the spleen and calming the liver, resolving phlegm and dispelling wind	*“Golden Mirror of Medicine” (醫宗金鑒)卷五十一*	Liu et al.* [32]
	(柴勺六君子湯)		健脾平肝, 化痰祛風		
15	Ge Gen Qin Lian Decoction	Puerariae Lobatae Radix, Scutellariae Radix, Coptidis Rhizoma, Glycyrrhizae Radix Et Rhizoma	Removal of external symptoms and regulation of internal functions	*“Treatise on Exogenous Febrile Disease”(傷寒論)*	Zhang et al.* [34]
	(葛根芩連湯)		解表清裡		
16	Chaihu Shu Gan Powder	Citri Reticulatae Pericarpium, Bupleuri Radix, Chuanxiong Rhizoma, Cyperi Rhizoma, Aurantii Fructus, Paeoniae Radix Alba, Glycyrrhizae Radix Et Rhizoma	Diversifying the liver, promoting blood circulation and relieving pain	*“Yi Xue Tong Zhi”(醫學統旨)*	Lei et al.* [31]
	(柴胡舒肝散)		疏肝理氣, 活血止痛		
17	Chai Hu Yin Chen Wu Ling Powder	Bupleuri Radix, Artemisiae Scopariae Herba, Plantaginis Seme, Akebiae Caulis, Polyporus, Cinnamomi Cortex, Poria, Alismatis Rhizoma, Atractylodis Macrocephalae Rhizoma	Lowering fire, removing blood stasis and eliminating fat, strengthening the spleen, promoting dampness and activating blood circulation	*“Jing Yue Quan Shu”(景嶽全書)*	Long et al.* [23]
	(柴胡茵陳五苓散)		降火祛瘀消脂, 健脾利濕活血		
18	Ling Gui Zhu Gan decoction (苓桂術甘湯)	Cinnamomi Ramulus, Atractylodis Macrocephalae Rhizoma, Glycyrrhizae Radix Et Rhizoma Praeparata Cum Melle, Poria	Warming Yang and Removing Dampness溫陽化飲, 健脾利濕	*"Synopsis of the Golden Chamber" (金匱要略)*	Wu et al.* [51]

^*^These RCTs adjusted the herbal composition of the formula based on the classical formula, i.e.…, increased or decreased the type or dosage of herbs

Sixty-two RCTs reported the history of NASH, the shortest history was 3 months and the longest was 16 years. Among the 72 RCTs that reported the type of visit, 47 trials enrolled outpatients, 21 trials enrolled both outpatients and inpatients, and 4 trials enrolled only inpatients. Regarding the patient source, 8782 participants in 81 RCTs were from a single hospital, and 1791 participants in 13 trials were from multiple hospitals. The mean sample size was 94 subjects, with sample sizes varying from 8 to 220 subjects.

#### Inclusion and exclusion criteria

The inclusion criteria and exclusion criteria of the 112 RCTs were shown in Additional file 1. To identify NASH patients, 103 RCTs used relevant standards or literature and established integrative medicine and western medicine diagnostic criteria. These researches considered age, medication history, biochemical indexes, alcohol intake, adherence, comorbidities, and disease history as inclusion criteria, in addition to diagnostic criteria.

Of the 112 RCTs, 91 reported exclusion criteria, while 21 did not specify their exclusion criteria (see Additional file 2). Comorbidities, established causes of fatty liver or liver disease, pregnancy, or breastfeeding were the primary exclusion factors. Furthermore, 29 RCTs considered the risk of complex herbal components causing allergies and excluded individuals who could have had such reactions or had a high sensitivity to the test drugs.

#### Comparator group setting

The medication of the control group could be divided into 6 types (as shown in Table 6). Chemical medicines were the most commonly used comparator, followed by TCMs and biological products. In addition, three RCTs employed behavioral interventions, such as like changes in eating or exercise habits, as the control group.

**Table 6:** Clinical research design status

Clinical research design Items	Including types	Trials number	Participants number
Participants enrollment			
Inclusion criteria	Including: diagnosis criteria, biochemical indexes, age, medication history, radiological examination, good adherence, comorbidities, liver histology test, no mention of inclusion criteria, history of NASH, not participating in other clinical trials, alcohol intake requirements	112	10,573
Exclusion criteria	Including: comorbidities, fatty liver or liver disease with an established cause, pregnancy or breastfeeding	91	8824
	Allergy, age, analogous therapeutic medication history, relevant drug usage history that might have an impact on the trial, non-cooperation		
	Excessive alcohol intake, genetic metabolic conditions, participation in other RCT, liver enzymes, poor adherence, drug abuse		
	Not specific the exclusion criteria	21	1749
Diagnostic criteria	Integration medicine and western medicine criteria	75	7622
	Western medicine diagnostic criteria	24	2036
	Integration medicine criteria	4	350
	Not mentioned	9	565
Country	China	108	10,341
	Iran	3	224
	Japan	1	8
Age	Range: 16–76 years old	107	10,070
History of NASH	Range: 3 months–16 years from	62	5806
	Not mentioned	50	4767
Visit type	Outpatients	47	4579
	Inpatients	4	326
	Inpatients or outpatients	21	1682
	Not mentioned: 40 trials		
Comorbidity	Obesity	2	169
	Type II diabetes	1	62
	COPD	1	60
	Hyperuricemia	1	59
	Chronic hepatitis B	1	68
	Glycometabolic disease (1 trial)	1	119
Source	Single hospital	81	8782
	Multiple hospitals	13	1791
Size	Mean: 94 ± 31 patients	\	\
	Range: 8 to 220 patients	\	\
Interventions			
Medication	Only TCMs	73	7347
	TCMs combining with other medicine	39	3226
Other interventions	Exercise and/or diet	80	7341
Comparator			
Medication	Chemical medicine	92	8691
	Chinese patent medicine	6	529
	TCMs and Chemical medicine	2	219
	Placebo	7	468
Exercise/diet		3	414
Other interventions	Basic treatment	1	32
No comparator	Self-comparison	1	220
Outcome	Primary outcome	\	\
	Overall clinical efficacy rate		
	Hepatic function outcomes		
	Blood lipid profiles		
	Radiological response improvement		
	Secondary outcome	\	\
	TCMs syndrome scores efficacy rate		
	Blood sugar		
	Liver fat, Indexes of inflammation and tumor necrosis factor		
	Other indicators		
	Adverse effect	51	5229
Research design	Single-arm	1	
	Two-arms	110	
	Three-arms	1	
	Time period < 6 months	90	8130
	Time period = 6 months	21	2375
	Time period = 12 months	1	68

#### Clinical outcomes

All the studies set up different efficacy detection systems, such as overall clinical efficacy rate, serum indicators, radiological response, etc. (Table 7). Based on the traditional Chinese medicine theory, the effectiveness of the TCMs prescriptions was mainly reflected in soothing liver qi stagnation (24 RCTs), reducing cellulite (15 RCTs), tonifying spleen (8 RCTs). After treatment, the test group can generally have a good improvement compared with that before treatment, but not all indicators showed intergroup differences. Accordingly, this study synthesised the changes in relevant indicators from the perspective of clinical outcomes (Table 8).

**Table 7:** The medications of comparator groups among 108 trials

Medications of comparator groups	Trials number
Chemical medicines	89
Polyene phosphatidylcholine capsules	42
Silybin meglumine tablets or granules	15
Tiopronin tablets	7
Ursodeoxycholic acid tablets	4
Diammonium glycyrrhizinate enteric-coated capsules	4
Reduced glutathione for injection	1
Reduced glutathione for injection + ursodeoxycholic acid tablets	1
TCMs (Chinese patent medicines)	6
Hugan tablets	3
Danning tablets	2
Yiganning tablets	1
Biological products	3
Group bifidobacterium triptans capsules	1
Bacillus licheniformis capsules	1
Bacillus subtilis duplex enteric capsules	1
Chinese patent medicine combined with chemical medicine	2
Dangfeiliganning capsules + Benzbromarone tablets	1
Fufangyiganling tablets + Polyene phosphatidylcholine capsules	1
Basic therapy (non-mention about specific drugs)	1
Placebos	7

**Table 8:** Effective clinical indicators

Effective indicators	Trials number	Patients number
Primary outcome		
Overall clinical efficacy rate	78	7075
Hepatic function outcomes	81	8650
Blood lipid profiles	81	7794
Radiological response improvement	34	3857
Secondary outcome		
Therapeutic effect index	11	1118
Blood sugar	9	801
Liver fat	7	654
Indexes of inflammation	5	424
Necrosis factor	1	120
Other indicators		
BMI	15	2044
Intestinal flora	3	212

#### Adverse effects

Adverse effects were reported in 51 RCTs and not mentioned in others. Twenty-eight RCTs reported no adverse effect was observed in either the test or control groups. The main adverse effects observed in the 23 remaining trials were related to the digestive system, including stomachache, diarrhea, nausea, and constipation. None of the reported adverse reactions were related to the test drug. Out of a total of 82 participants who experienced side effects, two of them experienced severe side effects, which were stroke and back pain. In the control group, one participant out of 55 subjects experienced a serious adverse reaction, acute appendicitis.

#### Efficacy assessment and case report of cure

There were 57 RCTs that reported the number of cure cases, and it was observed that different efficacy assessment criteria were used to determine a cure. Specifically, 13 RCTs based their cure criteria on changes in TCMs syndrome, while 43 RCTs relied on overall clinical efficacy.

The efficacy assessment criteria of TCMs syndrome were referred from guidelines and Chinese expert consensus, including Consensus Opinions on the Diagnosis and Treatment of NALFD with TCMs and Western Medicine (2011 edition [131], 2017 edition [132]), Chinese Medicine Clinical Research of New Drugs Guiding Principles (2002 edition [17]), Guidelines for Management of NAFLD (2010 edition[133]), Diagnostic Efficacy Criteria for TCMs Diseases (1994 edition [134]). The primary criteria for determining cure were a therapeutic effect index greater than 90% or 95% (as shown in the Additional file 3).

The criteria for overall clinical efficacy were primarily based on "Consensus opinion on the combined Chinese and Western medicine treatment of non-alcoholic fatty liver disease," "Relevant standards for the Chinese medicine treatment of non-alcoholic fatty liver disease," "guidelines for clinical research on new Chinese medicine 2002," "guidelines for the prevention and treatment of non-alcoholic fatty liver disease," and "guidelines for the treatment of Chinese medicine digestive diseases". Of the 57 RCTs, 11 trials did not specify the basis for their cure criteria setting, and three trials referred to the efficacy criteria in the literature (see Additional file 4).

## Discussion

TCMs has been widely used to manage liver diseases for thousands of years, especially in China [135]. While TCMs does not explicitly describe NASH, its symptoms can be classified according to traditional TCMs categories, such as "hypochondriac pain", "accumulation", and "phlegmatic mass", which are often linked to factors such as improper diet, physical exhaustion, and poor mental health [11, 136]. Accrodingnally, the treatment principle for NASH is improving variety symptoms, such as blood stimulation and phlegm emission, spleen tonification and qi management, liver cleaning, and bile secretion stimulation [136], by the major mechanisms of changing cholesterol levels, enhancing liver function, reducing liver fibrosis, and influencing gut flora [12].

This research found evidence supporting the efficacy of herbal compounds or single herbal extracts in treating NASH based on included RCTs. At present, no approved therapy method for NASH exists, and the development of TCMs drug therapies and research may contribute to the discovery of novel therapeutic modalities [137].

### About the TCMs medication

This study discovered that there are many applications of herbal formulas for treating NASH, including Chinese traditional prescriptions, classic Chinese traditional prescriptions, or self-prepared TCMs prescriptions. Currently, some studies indicated that herbal formulas can regulate intestinal flora, which in turn reduces liver inflammation and oxidative stress, achieves regulation of individual immunity, ultimately lower serum ammonia levels, improves lipid metabolism, protects the intestinal barrier, and reverses liver fibrosis with TLR4 signaling pathway may be closely related [138].

Twenty-one RCTs included in this study were conducted clinical studies on 18 Chinese traditional prescriptions. The possible mechanism of action of Yin Chen Hao Decoction may be to control liver fat by enhancing lipocalin and endothelial progenitor cells, which in turn regulate peroxisome proliferator-activated receptor γ (PPAR γ) for the treatment of liver fat disease [139]. PPAR γ can encourage the absorption and storage of FFA in adipose tissue and are thought to be a key target for the treatment of fat liver [140, 141]. In addition to regulating PPAR γ, Ge Gen Qin Lian Decoction may also be used to treat liver fat diseases via reducing LDL and HDL [142]. Ling Gui Zhu Gan Decoction [143] and Huang Lian Jie Du Decoction [144] both can lower TC, TG, and LDL-C levels in order to reduce liver fat.

Noteworthy, the RCTs included in this study referred to some classic Chinese traditional prescriptions such as “Yi Guan Decoction”(YGD) [74], “Ling Gui Zhu Gan Decoction”(LGZGD) [51], “Huang Lian Jie Du Decoction” (HLJDD) [26, 49] and “Yin Chen Hao Decoction” (YCHD) [47, 56, 65, 73]. Chinese medicine's multi-component and multi-mechanism characteristics form the basis on which it can be applied to treat various diseases [145, 146]. YGD is well known for its hepatoprotective properties [147, 148]. Especially its antifibrotic effects through preventing hepatic stellate cell activation and hepatocyte death [149], antiangiogenic effects via the HIF-1/VEGF signaling pathway [150], and anti-inflammation [151]. HLJDD has several functions in treating liver diseases, including liver protection [152, 153], anti-inflammatory[154], blood sugar[155, 156] and lipidmanagement [157, 158]. LGZGD and YCHD have also shown their effectiveness in treating non-alcoholic fatty liver disease or liver fibrosis [159, 160].

The "Catalogue of Classical TCMs Prescription (the First batch)" (CCTCMP), published in 2018 by the National Administration of Traditional Chinese Medicine, advocats for and promots the drug development of Chinese herbal formulas [161]. Some trials in this study used formulas from CCTCMP, including "Ling Gui Zhu Gan Decoction [51]," "Yi Guan Decoction [74]," and "Dang Gui Bu Xue Decoction [54, 59, 64, 101, 124]". Chinese regulatory authorities have continuously promoted the CCTCMP, and new drug development in NASH based on CCTCMP may be carried out to accelerate the launch of new medications within the framework of the national strategy of active promotion.

Herbs have pharmacological effects that are significantly stronger when administered together (in herb-pairs) than when taken alone [162]. Network analysis showed that "Salviae Miltiorrhizae Radix Et Rhizoma and Alismatis Rhizoma" and "Salviae Miltiorrhizae Radix Et Rhizoma and Bupleuri Radix" were closely connected drug-pair. Preclinical research validated the efficacy for treating liver disease of Salviae Miltiorrhizae Radix Et Rhizoma [163], Alismatis Rhizoma [164] and Bupleuri Radix [165]. Exploring the treatment of NASH with TCMs by selecting optimal drug-pair for pharmacological action studies or clinical selection of drug pairs to treat patients with numerous comorbidities can be valuable, particularly by researching medication pairings with a high frequency of occurrence.

### About the clinical trial design

The included studies were primarily single-centre clinical trials with limited sample numbers, short durations, and varying inclusion and exclusion criteria. A significant issue was a lack of standardised clinical design and inadequate information reporting. Firstly, the diagnostic criteria were rather inconsistent. Some of the studies did not completely adhere to the recommendations' criteria or did not properly cite the diagnostic criteria. Only four studies cited the recommendations from NAFLD guidelines. Secondly, no study had a clear description of the staging of the included patients, which is also a non-standard aspect of the existing clinical trials. Most clinical outcome observation indicators were liver enzymes, blood lipids, or B-ultrasound results, with only two trials reporting liver biopsy results [20, 122]. Furthermore, most investigations lasted less than 6 months, with only two studies having a 3-month follow-up [86, 111]. It is important to note that liver biopsy is the gold standard for diagnosing NASH, and the majority of studies did not specify the basis for diagnosis[166]. According to Filozof’s [167] and Sanyal’s[168] researches, the diagnosis of NASH could be made based on the biopsy findings, and the severity of the disease should be established at the time of inclusion. Excluding early validation trials, the clinical trial period should go longer than 6 months and a 6-month follow-up period [162].

Although 108 RCTs indicated significant efficacy differences between the test and control groups, the clinical design limitations may constrain the representativeness of the relevant results. The definition of cured cases was unclear, and changes in clinical indications before and after treatment cannot be used to determine a cure. Additional clinical research with larger samples, multicentre studies, extended durations, and standardised treatments is necessary to establish the efficacy of TCMs in treating NASH [12].

There were several limitations in our study. The medication analysis did not explore each Chinese herbal formula's mechanisms or active compounds. The results of overall Chinese herbal formulas did not differentiate between "only TCMs" and "TCMs combined with Western medicine" interventions. Moreover, all RCTs were included without quality evaluation, making it difficult to determine the efficacy and safety of TCMs for NASH. Future clinical trials of TCMs for NASH should be conducted with greater rigor, and mechanistic fundamental research needs further investigation.

## Conclusion

The rising incidence, related health burden, and absence of authorised medication for patients with NASH create a significant unmet medical need. NASH can be treated with Chinese medicine on a solid and long-term clinical basis. The new drug development of TCMs based on traditional Chinese classical prescriptions may facilitate the quick development of novel treatment modalities and the quick commercialisation of pharmaceuticals, considering the national support policies. Additionally, the pharmacological analysis of herbal remedies for NASH may benefit from investigating highly frequent drug pairs. TCMs for NASH has been the subject of previous RCTs yet these studies were still flawed or inconsistent. To get more convincing evidence for using TCMs to treat NASH, follow-up research should improve the clinical trial design.

## Supplementary Information

**Additional file1. Table S1**: Inclusion criteria of the included studies.

**Additional file 2. Table S2**: Exclusion criteria of the included studies.

**Additional file 3. Table S3**: Efficacy criteria of TCM syndrome efficacy and cure cases reports.

**Additional file 4. Table S4**: Diagnostic criteria and cure of overall clinical efficacy.

## Data Availability

All data are fully available without restriction.

## Abbreviations

ADP: Adenosine diphosphate
ALT: Alanine aminotransferase
AST: Aspartate aminotransferase
CAP: Controlled attenuation parameters
FFA: Free fatty acids
FINS: Fasting plasma insulin
FPG: Fasting plasma glucose
GGT: Gamma-glutamyl-transpeptidase
HDL: High density lipoprotein-cholesterol
LDL: Low density lipoprotein-cholesterol
L/S ratio: Liver to spleen ratio
PPAR-α: Peroxisome proliferators-activated receptors
TC: Total cholesterol
TG: Triglyceride
TNF-α: Tumor necrosis factor-α
UA: Uric acid
WM: Western medicine
