# Supplementary material for: Systematic analysis of randomised controlled trials of Chinese herb medicine for non-alcoholic steatohepatitis (NASH): implications for future drug development and trial design
Source: Chin Med. 2023 May 19;18:58. doi: 10.1186/s13020-023-00761-5 (PMC10199512; doi:10.1186/s13020-023-00761-5)
Supplement: Supplementary file 1 — Additional file1. Table S1: Inclusion criteria of the included studies. [file 13020_2023_761_MOESM1_ESM.docx]

Additional file 1: Table 1. Inclusion criteria of the included studies

| **No.** | **Inclusion criteria** | **Contents** | **Trials number** |
| --- | --- | --- | --- |
| 1 | Diagnosis criteria | Integrative medicine and Western medicine | 74 |
|  |  | Western medicine | 29 |
|  |  | Not specific the diagnosis criteria | 9 |
| 2 | Age | 18-65 years old Other age range | 31  26 |
|  |  | Not specific the age requirement | 55 |
| 3 | Medication history | Analogous therapeutic medication history: never; within 2 weeks, 4 weeks or 8 weeks | 25 |
| 4 | Biochemical indexes | liver function indicators, blood lipids, blood sugar, body weight | 15 |
| 5 | Radiological examination | B-ultrasound, computed tomography | 10 |
| 6 | Alcohol intake requirements | No alcohol No more than 140 g /week for men and less than 70 g /week for women No more than 20 g/day | 7 |
| 7 | Adherence | Good compliance, can cooperate to complete the test | 5 |
| 8 | Comorbidities | There are no other diseases such as heart, brain, or kidney disease, nor are they combined with other liver diseases. | 5 |
| 9 | Liver histology test | Liver histology test | 4 |
| 10 | History of NASH | Over 6 months of NASH | 1 |

NASH: non-alcoholic steatohepatitis
