# Supplementary material for: Systematic analysis of randomised controlled trials of Chinese herb medicine for non-alcoholic steatohepatitis (NASH): implications for future drug development and trial design
Source: Chin Med. 2023 May 19;18:58. doi: 10.1186/s13020-023-00761-5 (PMC10199512; doi:10.1186/s13020-023-00761-5)
Supplement: Supplementary file 2 — Additional file 2. Table S2: Exclusion criteria of the included studies. [file 13020_2023_761_MOESM2_ESM.docx]

Additional file 2: Table 2. Exclusion criteria of the included studies

| **No.** | **Exclusion criteria** | **Contents** | **Trials number** | |
| --- | --- | --- | --- | --- |
| 1 | Comorbidities | Hemochromatosis, thyroid disorders, biliary obstruction, heart, lung, and kidney illnesses, mental disorders, endocrine disorders, hyperlipidemia, hematopoietic system disorders, and immune system disorders, etc. | | 69 |
| 2 | Fatty liver or liver disease with an established cause | Cirrhosis, viral hepatitis, decompensated liver disease, autoimmune liver disease, and other fatty liver diseases, etc. | | 66 |
| 3 | Pregnancy or breastfeeding | Women during pregnancy | | 62 |
| 4 | Allergy | Allergies or intolerance to the test-drug substances | | 29 |
| 5 | Age | Age range that does not match the inclusion criteria | | 24 |
| 6 | Analogous therapeutic medication history | Use of TCM or Western medicine for NASH before the start of the trial | | 22 |
| 7 | Relevant drug usage history that might have an impact on the trial | Use of medications that might have an impact on the study, such as comorbidity medications, diet supplements, immunomodulatory medications, etc. | | 13 |
| 8 | Non-cooperation | Not administering medications as prescribed, incomplete follow-ups | | 12 |
| 9 | Excessive alcohol intake | Excessive alcohol intake, such as alcohol abuse | | 12 |
| 10 | Genetic metabolic conditions | α1-antitrypsin deficiency, hepatolenticular degeneration, and β-lipoprotein deficiency, etc. | | 10 |
| 11 | Participation in other RCTs | participated in another study within three months of the trial's start date or are currently a participant in another trial | | 10 |
| 12 | Liver enzymes | ALT, AST, GGT, and other liver function indicators are 2.5 times or 5 times higher than the normal value | | 9 |
| 13 | Poor adherence | Require whole gastrointestinal parenteral feeding or have severe gastrointestinal issues that make it hard to take medicines routinely. | | 7 |
| 14 | Drug Abuse | History of drug abuse | | 3 |

TCM, Traditional Chinese Medicine; NASH, non-alcoholic steatohepatitis; ALT, Alanine aminotransferase; AST, Aspartate aminotransferase; GGT, Gamma-glutamyl-transpeptidase.
