# Supplementary material for: Systematic analysis of randomised controlled trials of Chinese herb medicine for non-alcoholic steatohepatitis (NASH): implications for future drug development and trial design
Source: Chin Med. 2023 May 19;18:58. doi: 10.1186/s13020-023-00761-5 (PMC10199512; doi:10.1186/s13020-023-00761-5)
Supplement: Supplementary file 3 — Additional file 3. Table S3: Efficacy criteria of TCM syndrome efficacy and cure cases reports. [file 13020_2023_761_MOESM3_ESM.docx]

Additional file 3: Table 3. Efficacy criteria of TCM syndrome efficacy and cure cases reports

| **No** | **Year** | **Authors** | **Number of** | **Cure cases** | **Curative response criteria** |
| --- | --- | --- | --- | --- | --- |
|  |  |  | **participants** |  |  |
| 1 | 2022 | Sun et al. [24] | 54 | T: C=4:3* | Clinical symptoms and signs disappeared completely，therapeutic effect index no less than 95% |
| 2 | 2022 | Tai et al. [25] | 80 | T: C=10:3* | Clinical symptoms and signs disappeared completely，therapeutic effect index no less than 95% |
| 3 | 2021 | Liu et al. [32] | 90 | T: C=14:5* | Clinical TCM symptoms and signs disappeared completely，therapeutic effect index no less than 95% |
| 4 | 2021 | Wang et al. [33] | 120 | T: C=33:19* | Clinical symptoms and signs disappeared completely，therapeutic effect index no less than 95% |
| 5 | 2021 | Jin [29] | 116 | T: C=35:20* | Clinical symptoms and signs disappeared completely，therapeutic effect index no less than 95% |
| 6 | 2021 | Lei et al. [30] | 100 | T: C=27:18* | Clinical TCM symptoms and signs disappeared completely，therapeutic effect index no less than 95% |
| 7 | 2019 | Lan et al. [38] | 90 | T: C=19:12* | Clinical TCM symptoms and signs disappeared completely，therapeutic effect index no less than 95% |
| 8 | 2019 | Zhou et al. [41] | 129 | T: C=20:11** | Therapeutic effect index no less than 95% |
| 9 | 2018 | Li et al. [46] | 140 | T: C=15:12** | Therapeutic effect index no less than 90% |
| 10 | 2018 | Wu et al. [51] | 72 | T: C=20:10* | Clinical TCM symptoms and signs disappeared completely，therapeutic effect index no less than 90% |
| 11 | 2015 | Feng et al. [77] | 80 | T: C=10:6** | Therapeutic effect index no less than 90% |
| 12 | 2013 | Li et al. [93] | 164 | T: C=20:14** | Therapeutic effect index no less than 90% |
| 13 | 2012 | Qian et al. [103] | 220 | 16 | Therapeutic effect index no less than 90% |

*p<0.05, **p<0.01. TCM, traditional Chinese medicine; CT, Computerized tomography
