# Supplementary material for: Systematic analysis of randomised controlled trials of Chinese herb medicine for non-alcoholic steatohepatitis (NASH): implications for future drug development and trial design
Source: Chin Med. 2023 May 19;18:58. doi: 10.1186/s13020-023-00761-5 (PMC10199512; doi:10.1186/s13020-023-00761-5)
Supplement: Supplementary file 4 — Additional file 4. Table S4: Diagnostic criteria and cure of overall clinical efficacy. [file 13020_2023_761_MOESM4_ESM.docx]

Additional file 4: Table 4. Diagnostic criteria and cure of overall clinical efficacy

| **No** | **Year** | **Authors** | **Number of participants** | **Cure cases** | **Curative response criteria** |
| --- | --- | --- | --- | --- | --- |
| 1 | 2022 | Long et al. [23] | 120 | T: C=8:0** | The results of lipid, ALT and AST returned to normal |
| 2 | 2021 | Lei et al. [31] | 80 | T: C=7:4* | Clinical symptoms and signs disappeared completely; liver function and blood lipids results returned to normal; B-ultrasound examination showed the disappearance of fatty liver |
| 3 | 2021 | Chen et al. [27] | 104 | T: C=28:9* | Clinical symptoms and signs disappeared completely; liver function and blood lipids returned to normal; B-ultrasound result showed no fatty liver |
| 4 | 2021 | Zhang et al. [34] | 62 | T: C=15:6* | B-ultrasound result showed no fatty liver; all laboratory indicators returned to normal |
| 5 | 2020 | Lin et al. [35] | 80 | T: C=5:2* | Clinical symptoms and signs disappeared completely; the results of liver function, lipids, FPG, UA, FINS, FFA, TNF-α, ADP, PPAR-α, and CAP returned to normal; liver/spleen CT ratio more than 1, therapeutic effect index no less than 95% |
| 6 | 2018 | Chen et al. [44] | 95 | T: C=17:3* | Clinical symptoms and signs disappeared completely; therapeutic effect index no less than 95% |
| 7 | 2018 | Fan et al. [45] | 88 | T: C=18:7** | Blood lipids and transaminases returned to normal, and B-ultrasound examination showed the disappearance of fatty liver signs |
| 8 | 2018 | Lin et al. [47] | 80 | T: C=16:10* | Clinical symptoms and signs disappeared completely; efficacy index more than 90% |
| 9 | 2018 | Mai et al. [50] | 80 | T: C=9:3* | When the level of fatty infiltration in the liver is in the normal range on ultrasound at 24 weeks |
| 10 | 2017 | Dai et al. [52] | 32 | T: C=3:1* | Clinical symptoms and signs disappeared completely，therapeutic effect index bigger than 95% |
| 11 | 2017 | Hu et al. [54] | 140 | T: C=38:30** | Clinical symptoms and signs disappeared completely; efficacy index more than 95％ |
| 12 | 2016 | Tang et al. [65] | 120 | T: C=21:17** | Clinical symptoms and signs disappeared completely; therapeutic effect index more than 90％; CT result showed that liver density returned to normal; ALT and AST returned to normal |
| 13 | 2016 | Tian et al. [66] | 100 | T: C=13:7* | Clinical symptoms and signs disappeared completely, therapeutic effect index no less than 90% |
| 14 | 2016 | Wang et al. [67] | 200 | T: C=56:32* | Clinical symptoms and signs disappeared completely; therapeutic effect index no less than 90%; the results of liver function, ALT, AST and GGT returned to normal |
| 15 | 2016 | Zhang et al. [70] | 104 | T: C=25:15** | Clinical symptoms and signs disappeared completely, therapeutic effect index no less than 90%; the results of liver function, lipids, B-ultrasound results returned to normal |
| 16 | 2015 | Ye et al. [85] | 46 | T: C=7:4* | Clinical symptoms and signs disappeared completely; The results of radiological response, liver function and blood lipids returned to normal |
| 17 | 2015 | Chen et al. [73] | 78 | T: C=15:10* | Clinical symptoms and signs disappeared completely; therapeutic effect index no less than 95%; ALT, AST, TC, TG and B-ultrasound results returned to normal |
| 18 | 2015 | Huang et al. [78] | 60 | T: C=3:0* | Clinical symptoms and signs disappeared completely; liver function returned to normal; B-ultrasound or CT examination results showed no fatty liver |
| 19 | 2015 | Li et al. [79] | 104 | T: C=12:3* | therapeutic effect index no less than 95%; clinical symptoms and signs disappeared completely; the results of ALT, TG and CT returned to normal |
| 20 | 2015 | Li et al. [80] | 124 | T: C=1:0** | therapeutic effect index no less than 95%; TC and TG returned to normal; B-ultrasound result showed no fatty liver |
| 21 | 2015 | Liu et al. [81] | 69 | T: C=7:4* | Clinical symptoms and signs disappeared completely; CT result showed that morphology and parenchyma of the liver returned to normal; liver function and blood lipids returned to normal |
| 22 | 2014 | Li et al. [87] | 60 | T: C=6:3* | Clinical symptoms and signs disappeared completely; liver function and blood lipids results returned to normal; B-ultrasound showed the regression of hepatic steatosis |
| 23 | 2014 | Yu et al. [89] | 100 | T: C=4:1* | Liver enzymes, blood lipids and B-ultrasound showed normalisation of liver morphology and parenchyma |
| 24 | 2013 | Qiu et al. [94] | 90 | T: C=14:6** | clinical symptoms and signs disappeared completely; liver function and blood lipids results returned to normal; B-ultrasound examination was better than before |
| 25 | 2013 | Wang et al. [95] | 80 | T: C=15:6* | Clinical symptoms disappeared completely; liver function, blood lipids returned to normal |
| 26 | 2013 | Zhan et al. [97] | 36 | T: C=8:5* | The results of radiological response assessed by B-ultrasound, liver function and blood lipids returned to normal |
| 27 | 2012 | Chen et al. [98] | 124 | T: C=31:27(p>0.05) | Clinical symptoms and signs disappeared completely; liver function and blood lipids results returned to normal; l L/S ratio was bigger than 1， or B-ultrasound results showed that the disappearance of fatty liver waveform |
| 28 | 2012 | Chen et al. [99] | 70 | T: C1:C2=6:5:0（p>0.05, p<0.05) | Clinical symptoms disappeared completely; therapeutic effect index no less than 95%; liver function returned to normal |
| 29 | 2012 | Ling et al. [100] | 62 | T: C=10:8* | Not mentioned |
| 30 | 2012 | Pei et al. [102] | 160 | T: C=13:9* | therapeutic effect index no less than 90%; liver function, blood lipids and B-ultrasound returned to normal |
| 31 | 2012 | Zhang et al. [106] | 60 | T: C=10:4* | Clinical symptoms disappeared completely; liver function, blood lipids and B-ultrasound or CT examination returned to normal |
| 32 | 2011 | Gu et al. [108] | 100 | T: C=24:16* | Therapeutic effect index no less than 95％ |
| 33 | 2011 | Zhang et al. [110] | 182 | T: C=29:12* | Liver function, blood lipids results and B-ultrasound or CT examination returned to normal; clinical symptoms disappeared completely or therapeutic effect index no less than 80％ |
| 34 | 2010 | Liang et al. [113] | 75 | T: C=21:10* | Therapeutic effect index no less than 95% |
| 35 | 2010 | Li et al. [112] | 65 | T: C=9:3* | Therapeutic effect index no less than 90%; B ultrasound showed no fatty liver; normal blood lipids and serum transaminases |
| 36 | 2010 | Ma et al. [115] | 80 | T: C=10:4** | Clinical symptoms and signs disappeared，the result of liver function and lipids return to normal, liver/spleen L/S ratio bigger than 1 |
| 37 | 2009 | Wang et al. [118] | 82 | T: C=7:3** | Clinical symptoms disappeared completely; liver function and blood lipids returned to normal; B-ultrasound showed that the liver echogenicity was normal in size and clear in outline with uniform distribution of intrahepatic light spots |
| 38 | 2009 | Zhang et al. [119] | 160 | T: C=34:8** | Clinical symptoms and signs disappeared completely; therapeutic effect index no less than 95%; liver function, blood lipids results and B-ultrasound examination returned to normal |
| 39 | 2008 | Lu et al. [122] | 80 | T: C=11:3** | Clinical symptoms disappeared completely；therapeutic effect index no less than 95 %; liver function returned normal; B-ultrasound examination showed the disappearance of fatty liver or L/S ratio bigger than 1 |
| 40 | 2008 | Sun et al. [123] | 61 | T: C=8:5* | B-ultrasound returned normal and TCM symptom disappeared |
| 41 | 2007 | Wang et al. [124] | 38 | T: C=10:4* | Clinical symptoms disappeared completely; liver function, liver fibrosis index and blood lipids results returned to normal range; radiological response returned to normal |
| 42 | 2007 | Yang et al. [125] | 128 | T: C=14:7* | Clinical symptoms and signs disappeared completely; liver function returned to normal; the reduction of TC should more than 20%, TG more than 40%,HDL more than 0.94nmol/L; B-ultrasound of the liver showed uniform intrahepatic dots and small vessels with mildly diminished deep hepatic echogenicity; liver/spleen CT ratio returned to normal |
| 43 | 2006 | Chen et al. [127] | 62 | T: C=6:3* | Clinical symptoms and signs disappeared completely，therapeutic effect index no less than n 95 %; liver function returned to normal |
| 44 | 2004 | Luo et al. [130] | 80 | T: C=27:6** | Clinical symptoms disappeared completely; the results of ALT and GGT returned to normal; the reduction of TC and TG more than 20%; B-ultrasound of liver shows that intrahepatic echogenicity was significantly diminished and post-hepatic attenuation is significantly reduced with clear intrahepatic vessels |

*****p<0.05, **p<0.01. TCM, Traditional Chinese medicine; CT, Computerized tomography; ADP, Adenosine diphosphate; ALT, Alanine aminotransferase ; AST, Aspartate aminotransferase; CAP, Controlled attenuation parameters; FFA, Free fatty acids; FINS, Fasting plasma insulin; FPG, Fasting plasma glucose; GGT, Gamma-glutamyl-transpeptidase; L/S ratio Liver to spleen ratio; PPAR-α, Peroxisome proliferators-activated receptors; TC, Total cholesterol; TG, Triglyceride; TNF-α, Tumor necrosis factor-α; UA, Uric acid; WM, Western medicine.
